# Effectiveness of low-level laser therapy on recovery from neurosensory disturbance after sagittal split ramus osteotomy: a systematic review and meta-analysis

**DOI:** 10.1186/s40902-020-00285-0

**Published:** 2020-12-17

**Authors:** Parsa Firoozi, Seied Omid Keyhan, Seong-Gon Kim, Hamid Reza Fallahi

**Affiliations:** 1 Faculty of Dentistry, Department of Oral and Maxillofacial Surgery, School of Dentistry, Zanjan University of Medical Sciences, Zanjan, Iran; 2 CMFRC, National Advance Center for Craniomaxillofacial Reconstruction, Tehran, Iran; 3 Craniomaxillofacial Research Center, Tehran University of Medical Sciences, Tehran, Iran; 4 Founder and Director of Maxillofacial Surgery and Implantology Research Foundation, Tehran, Iran; 5 Department of Oral and Maxillofacial Surgery, College of Dentistry, Gangneung-Wonju National University, Gangneung, 28644 Republic of Korea; 6 Dental Research Center, Research Institute of Dental Sciences, Shahid Beheshti University of Medical Sciences, Tehran, Iran

**Keywords:** Sagittal split ramus osteotomy, Low-level laser therapy, Inferior alveolar nerve, Photobiomodulation therapy

## Abstract

**Background:**

Orthognathic surgery such as bilateral sagittal split ramus osteotomy (BSSRO) for the treatment of mandibular deformities is one of the most common procedures in maxillofacial operations that may lead to neurosensory disturbance. In this study, we aimed to evaluate the effectiveness of low-level laser therapy (LLLT) on augmenting recovery of neurosensory disturbance of inferior alveolar nerve (IAN) in patients who underwent BSSRO surgery.

**Methods:**

A comprehensive literature search was conducted by two independent authors in PubMed, Cochrane Central Register of Controlled Trials (CENTRAL), Scopus, Embase, and Google Scholar electronic databases. Besides, a manual search of all textbooks and relevant articles were conducted. Searches took place in August 2020 and were limited to published and peer-reviewed articles from 2000 to 2020. All analysis was performed using the comprehensive meta-analysis (CMA) and the STATA MP (version:16) software. The weighted mean difference (WMD) using the inverse variance method and the standard mean difference (SMD) was considered for continuous variables.

**Results:**

Seventy-four papers were retrieved after removing duplicate studies and finally, eight studies were assessed for qualitative synthesis and five for meta-analysis. Totally, 94 patients were included in the meta-analysis. Based on the meta-analysis, it was shown that LLLT was not effective in a short interval (0 to 48 h) after surgery, but in a period of more than 1 month after surgery, the positive results of treatment can be observed strikingly. Also, LLLT side/group showed no significant difference in some aspects of neurosensory recovery such as thermal sensation compared to the placebo side/group.

**Conclusions:**

The meta-analysis of randomized controlled trials revealed that LLLT generally improves IAN sensory disturbance caused by BSSRO. Further high-quality clinical trials with longer follow-up periods and larger sample sizes are recommended.

## Background

Bilateral sagittal split ramus osteotomy (BSSRO) is a technique of orthognathic surgery that is utilized to adjust excess, deficiency, and asymmetry of the mandible [1]. The sagittal split osteotomy was described by Trauner and Obwegeser and then modified by DalPont and Epker et al. [2]. Despite its versatility and numerous advantages, various complications have been stated intermittently among studies. The most-reported complications include bad splits (pooled:2.3%), postoperative infection (pooled:9.6%), removal of osteosynthesis material (pooled:11.2%), and neurosensory disturbances (pooled:33.9%) [3]. As shown, neurosensory disturbance (NSD) is one of the most common complications of the bilateral sagittal split ramus osteotomy. Quality of materials, postoperative care, surgeons’ skills, and surgical time are correlated with the aforementioned complications [4].

Several factors have been reported to increase the incidence of nerve injury with BSSRO, namely, dissection of the soft tissues on the medial aspect of the mandibular ramus, large mandibular advancements, lateral course of the inferior alveolar nerve, long mandibular angle, and mechanical damage of the sensory fibers of the IAN during surgery [5, 6]. The reported symptoms of a nerve injury include paresthesia, dysesthesia (burning, stinging, or stabbing sensations), sensory deficits, allodynia, or hyperesthesia [7, 8]. Paresthesia is commonly observed, as also as hypoesthesia [9, 10]. Hyperesthesia and allodynia are less common [10, 11].

These symptoms may seriously affect a patient’s daily activities, such as drinking and eating, and may also lead to traumatic biting of soft tissues (lips or cheeks) during mastication. Also, some patients may experience severe pain that can be debilitating [12]. Therefore, it is reasonable to manage neurological complications. Although neurosensory recovery usually occurs spontaneously at some point after the nerve damage, additional methods can be utilized to improve and accelerate the healing process [13].

Several treatment modalities regarding the management of NSD are available. Follow-up observation (no treatment), medication, physiotherapy, local electrical stimulation, stellate ganglion block (SGB), acupuncture, low-level laser therapy (LLLT), and microsurgical repair are among the most common treatment modalities [14–16]. In recent years, several clinical trials on LLLT have shown a significant nerve function improvement [13, 17–20]. It has been demonstrated that LLLT induces modulatory effects on cells and tissues through non-thermal or non-ablative mechanisms [21–24]. Also, the proliferation, formation of granulation tissue, decrease of inflammatory cell count, angiogenesis stimulation, and increased collagen synthesis are the biological effects of LLLT [25].

In clinical conditions, low power lasers are generally applied to reduce pain, accelerate the inflammatory process, and enhance the healing rate of damaged tissues [26–28]. This meta-analysis was aimed to investigate the effectiveness of LLLT on augmenting recovery of neurosensory disturbance of inferior alveolar nerve in patients who underwent BSSRO surgery.

## Methods

### Protocol and registration

This study was organized based on the Preferred Reporting Items for Systematic Reviews and Meta-Analysis (PRISMA) guidelines [29]. We registered our review protocol at PROSPERO (CRD42020205952).

### Eligibility criteria

The present research aimed to answer the following question: Is low-level laser therapy effective to recover neurosensory impairment of the inferior alveolar nerve in patients who underwent BSSRO surgery?

We utilized PICOS components to define the research question: population (patients submitted to BSSRO surgery), intervention (low-level laser therapy before or after surgical procedure), comparison (other side of lower jaw as placebo, control group of participants who underwent BSSRO), outcome (laser therapy results in the improvement of neurosensory disorders), and study design (randomized controlled trials). No language limitation was considered to decrease the risk of bias. Studies were excluded if they (I) were unpublished articles (II) were non-peer-reviewed articles (III) were conference papers, editorial papers, and review articles (IV) had insufficient data.

### Information sources and search

Studies in this systematic review were selected via a systematic searching in the following electronic databases: PubMed, Cochrane Central Register of Controlled Trials (CENTRAL), Scopus, Embase, and Google Scholar. All keywords were checked with the MeSH (Medical Subject Headings) database and then were used. The search strategy in databases is presented in Table 1. Searches took place in August 2020 and were limited to published and peer-reviewed articles from 2000 to 2020. Also, the reference lists of all primary studies were searched manually for additional relevant publications.

**Table 1:** Full search strategy for electronic databases

**Searched phrase**	(Low-level laser OR low-level laser therapy OR laser OR phototherapy OR photodynamic therapy) AND (Inferior alveolar nerve OR alveolar nerve OR mandibular nerve OR trigeminal nerve) AND (sagittal split ramus Osteotomy OR bilateral sagittal split ramus osteotomy OR sagittal split ramus OR orthognathic surgery OR osteotomy)

### Study selection

All records were imported into the EndNote software (version X9.2), and duplicate studies were removed. Two reviewers pre-screened the titles and abstracts of the studies independently. Articles not meeting inclusion criteria and were irrelevant for the study were excluded. Disagreement about eligibility and any controversies between the two reviewers resolved through a discussion. Full text of retrieved studies were obtained and two authors evaluated the full-text articles based on inclusion criteria. Irrelevant articles according to title, abstract, and body text were excluded.

### Data extraction

The data extraction was performed by one author and checked by the second reviewer. The following data were tabulated: research design, sample size, age of participants (years), post-treatment and post-operative follow-up period, type of surgery, wavelength, energy density, time of laser application, total number of therapeutic sessions, and data related to neurosensory tests. Also, we contacted the authors of some studies if their data was insufficient for meta-analysis. We used WebPlotDigitizer online tool (Available from https://automeris.io/WebPlotDigitizer/) to extract appropriate data from charts if authors of a study did not respond to our emails.

### Risk of bias

Two reviewers independently evaluated the risk of bias in this research using Cochrane Collaboration’s assessment tool [30]. Seven domains of bias were evaluated: (1) random sequence generation, (2) allocation concealment, (3) blinding of participants and personnel, (4) blinding of outcome assessment, (5) incomplete outcome data, (6) selective reporting, and (7) others (follow-up period) [30].

### Data analysis

A meta-analysis was performed on two main neurosensory tests (2-point discrimination test and General VAS for sensitivity test) due to their sufficient data for analysis. All analysis was performed using the Comprehensive Meta-Analysis (CMA) and the STATA MP software (version:16) to determine pooled effects. For continuous variables, the weighted mean difference (WMD) using the inverse variance method was considered if the data were numerically similar. Otherwise, the standard mean difference (SMD) was considered.

All the results of the treatment were presented using 95% CI. *P <* 0.05 was considered for a significant difference. Heterogeneity was tested using *I*^2^ statistics. *I*^2^ < 50% at the level of *α* = 0.10 indicated a lack of heterogeneity across the studies. The fixed-effect model was used if the *P* value related to the heterogeneity was more than 0.10. Otherwise, the random-effects model was considered. All the *P* values were two-sided and the statistical significance was defined at level of *α* = 0.05.

## Results

### Study selection

**Fig. 1:**
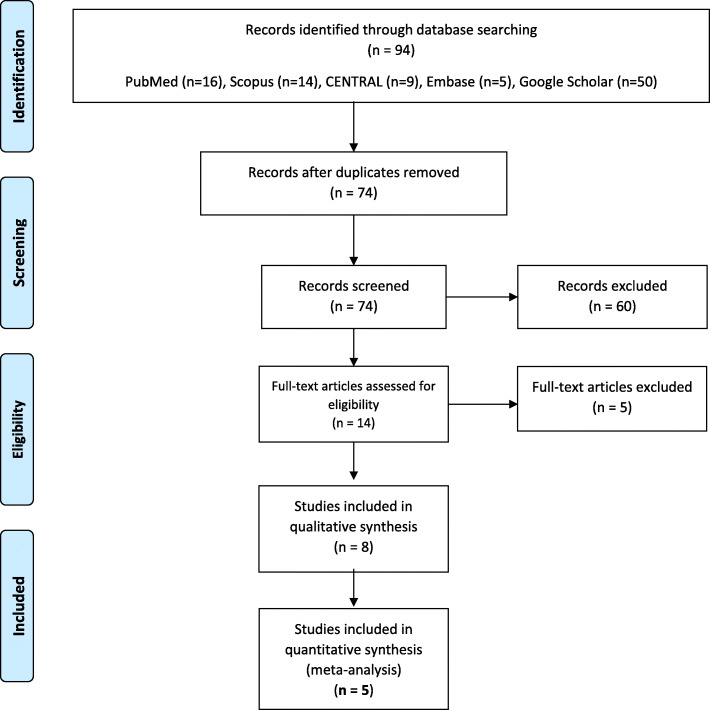
Algorithm showing retrieval of papers

With a comprehensive search, 74 papers were retrieved after removing duplicate studies. Titles and abstracts screened based on inclusion and exclusion criteria and 14 studies were retrieved and assessed for full-text evaluation based on the pre-determined inclusion and exclusion criteria and the assumed research question. Hence, five studies were excluded, and finally, eight studies were assessed for qualitative synthesis and five for meta-analysis [13, 17–19, 31–34] (Fig. 1).

### Study characteristics

All studies were randomized controlled trials. Six studies were double-blind, one was single-blind and one was triple-blind randomized controlled trials. Also, four studies had the split-mouth design in which one side of the lower jaw was radiated by laser and the other side subjected to LLLT without radiation as placebo [13, 18, 31, 34] and four had two separate treatment (test) and control (non-treated) groups [17, 19, 32, 33]. The latest study was conducted in 2020. Totally, 188 patients were evaluated; however, 94 patients were included in the meta-analysis. The study of Guraini et al. had the most post-treatment follow-up period and the study carried out by Buysse Temprano et al. had no post-treatment follow-up period [19, 31].

In all investigations, the diode laser (GaALAs) was used. In one research, a mix of laser and LED [33], in another study, a combination of two laser wavelengths [18], and in a third study, a combination of three laser wavelengths was used [13]. Also, intra-orally or intra/extra-orally were the lasers used. The typical points of laser radiation in the studies were osteotomy site mucosa, labial mucosa, buccal mucosa, and mandibular foramen in the intra-oral approach [13, 18, 31, 33], and in the extra-oral approach [13, 18, 31, 33], the skin surface along with the IAN pathway and chin were radiated. At least 3 [17] to 25 points [31] were radiated in the abovementioned areas (Table 2).

**Table 2:** Summary of included studies

First author/year	Design	Treatment/control group	Split-mouth design	Mean age (years) control/treatment	Type of surgery	Follow-up period after surgery (month)	Follow-up period after LLLT (month)	Wavelength (nm)	Energy (J) /energy density (J/cm^**2**^)	Power (mw)	Period of application (sec per point)	Total number of LLLT sessions	Days of LLLT (post-operation)	
			Treatment side/control side											
Führer-Valdivia 2014 [17]	DB-RCT	16/14		21.5 (8)/23 (5)	BSSRO	6	5	810 ± 20	9/32	100	90	8	1, 2, 3, 5, 10, 14, 21, 28	
Gasperini 2014 [18]	DB-RCT		10/10	30/30	BSSRO	2	1	660-789-780	1.2/5	20-60-70	10-20-40	14	0, 1, 2, 3, +10 days with 48 h intervalª	
Mohajerani 2017 [33]	DB-RCT	10/10		22.8 ± 3.6/24.1 ± 4.6	BSSRO	6	5	810-632	−/5 −/2		90	6	1, 2, 3, 7, 14, 28	
Eshghpour 2017 [13]	DB-RCT		16/16	23.1 ± 4.4/23.1 ± 4.4	BSSRO	2	1	660-810	2/1.5 2/7	200	10	9	1, 2, 3, +6 days in 3 weeks	
Guarini 2017 [19]	DB-RCT	33/9		29.8 (24)/25.8 (22)	BSSRO	24	23	810 ± 20	9/31.8	100		8	1, 2, 3, 5, 10, 14, 21, 28	
Buysse Temprano 2017 [31]	SB-RCT		12/12	30 /30	BSSRO	1	0	808	2.8/100	100	28	10		
Esmaeelinejad 2018 [32]	DB-RCT	20/20		27.35 ± 3.35/25.7 ± 4.06	BSSRO	13	12	810	−/8.4	70	60	10	0, 1, 2, 3, +6 days in 2 weeksª	
Sharifi 2020 [34]	TB-RCT		18/18	23 ± 5/23 ± 5	BSSRO	2	1	980	−/12	100	60	7	−1, 1, 3, 7, 14, 21, 28ª	

*DB-RCT* Double-blind randomized controlled trial, *SB-RCT* Single-blind randomized controlled trial, *TB-RCT* Triple-blind randomized controlled trial, *BSSRO* Bilateral sagittal split ramus osteotomy, *LLLT* Low-level laser therapy, a: 0 = immediately after surgery, −1: a day before surgery

### Risk of bias within studies

**Fig. 2:**
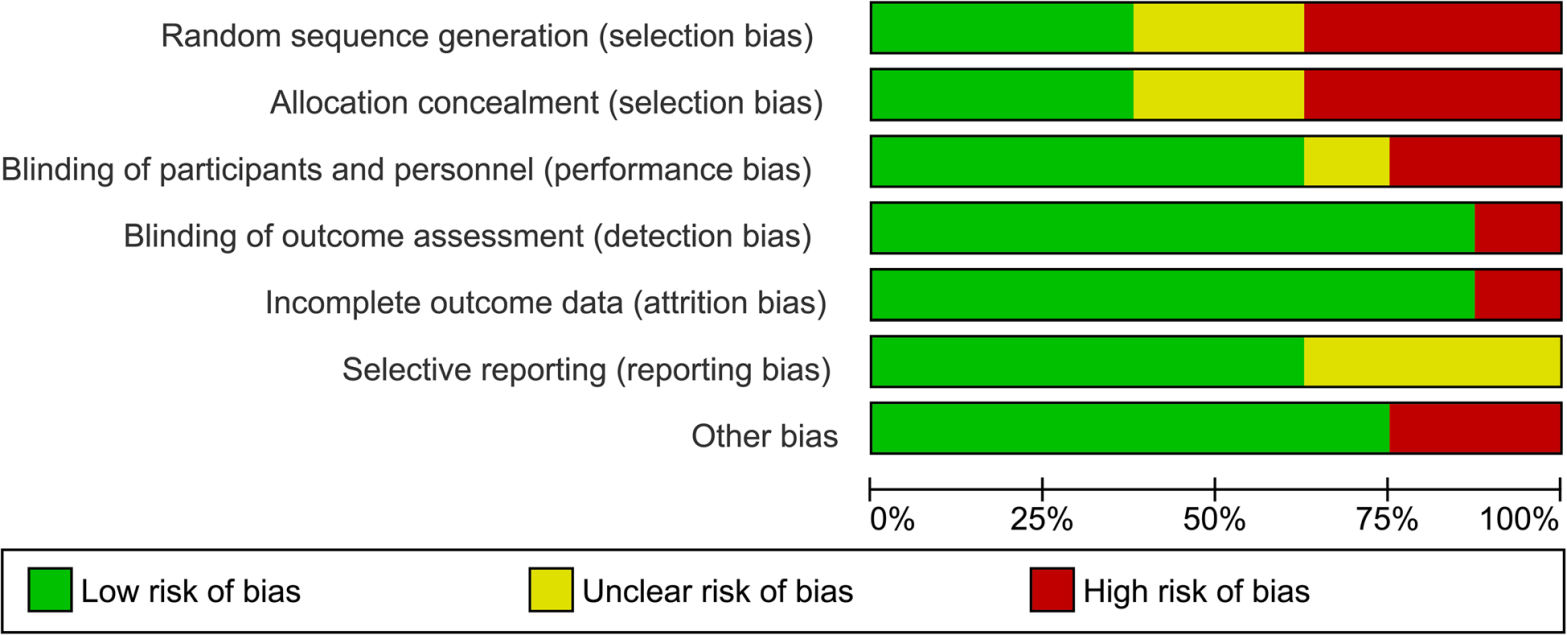
Risk of bias graph

**Fig. 3:**
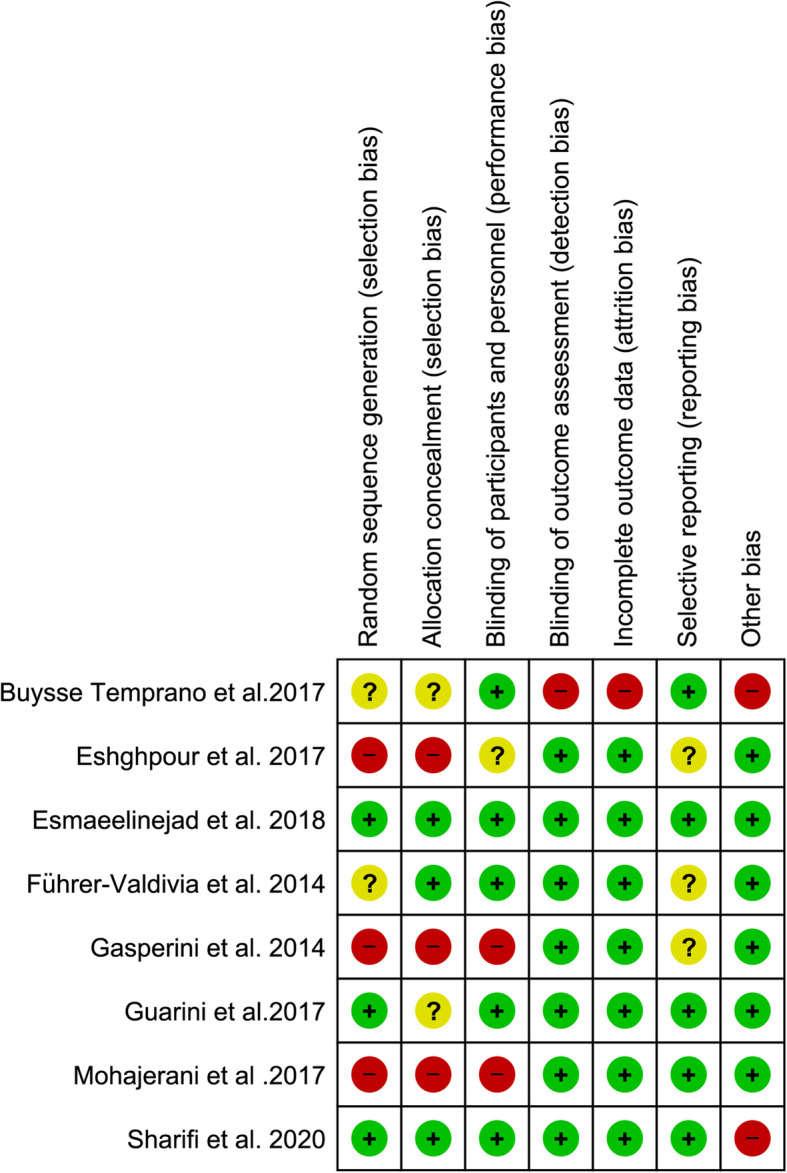
Risk of bias summary

Figures 2 and 3 present the risk of bias within studies. As is presented in the risk of bias graph (Fig. 2), the most bias within studies was related to selection bias (more than 50%). On the other hand, detection bias and attrition bias were minimal (less than 25%). Also, Buysse Temprano et al. [31] and Esmaeelinejad et al. [32] had the most and the least risk of bias among included studies, respectively (Fig. 3).

### Two-point discrimination test

**Fig. 4:**
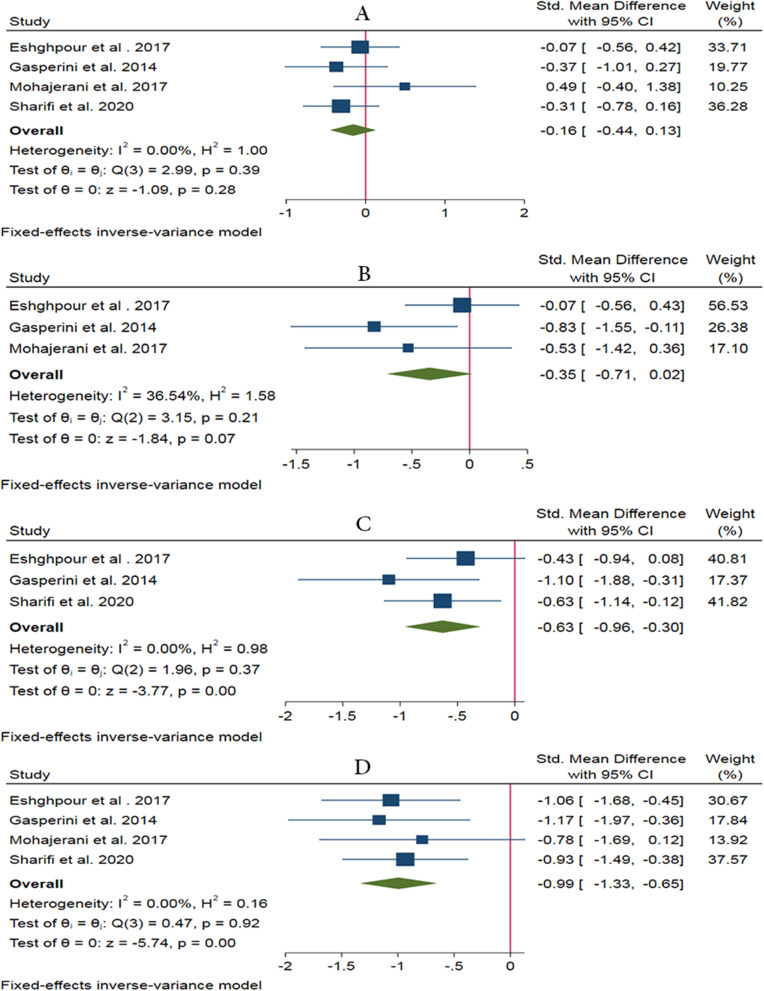
Forest plots of two-point discrimination test showing the effectiveness of LLLT in a short period (0 to 48 h) (**a**), after 2 weeks (**b**), 1 month (**c**), and 2 months after BSSRO (**d**)

Four studies have evaluated the effectiveness of LLLT in a short period of time (immediately after to 48 h after surgery) using the 2-point discrimination neurosensory test [13, 18, 33, 34]. In this test, a patient perceives the distance between the two touch stimuli with a relatively sharp tip on the skin surface. Shorter discrimination from a distance means stronger sensory strength. The analysis shows that the intervention is not effective in a short period (0 to 48 h) after BSSRO (SMD −0.16, 95% CI: −0.44 ~ 0.13, *I*^2^ = 0%, no heterogeneity). Also, three studies have evaluated the effectiveness of about 2 weeks after BSSRO [13, 18, 33]. The result of the analysis shows the lack of effectiveness in this period (SMD −0.35, 95% CI: −0.71 ~ 0.02, *I*^2^ = 36.54%, low heterogeneity). Additionally, two more analyses show a significant positive effect of LLLT on NSD recovery at 1 month (SMD −0.63, 95% CI: −0.96 ~ −0.30, *I*^2^ = 0%, no heterogeneity) and 2 months (SMD −0.99, 95% CI: −1.33 ~ −0.65, *I*^2^ = 0%, no heterogeneity) after BSSRO, respectively. Studies have also shown a significant difference in the LLLT group/side compared to the placebo group/side in time points over 2 months (approximately 6 to 24 months) after surgery based on the 2-point discrimination test (*P* < 0.05) [19, 32, 33] (Fig. 4).

### General sensitivity test

**Fig. 5:**
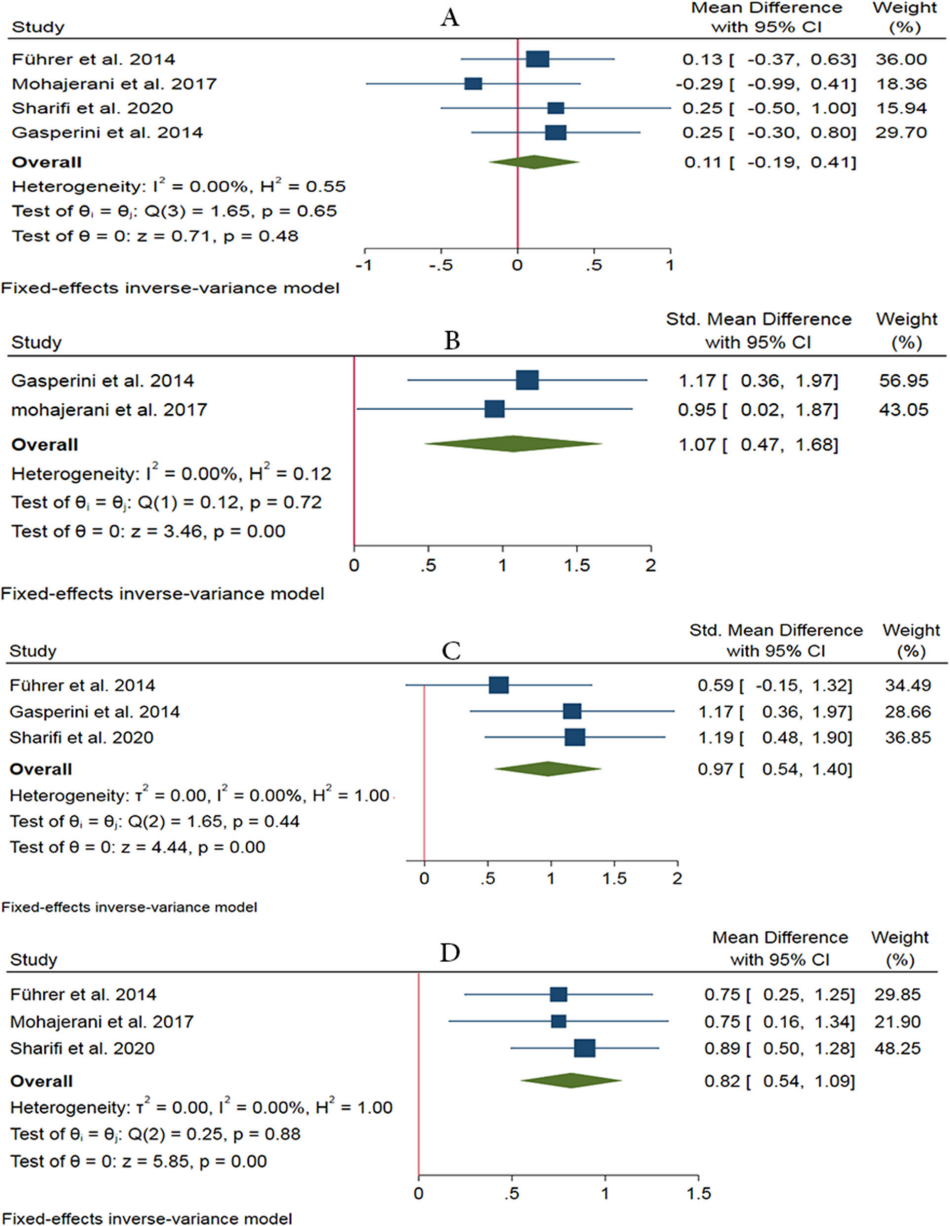
Forest plots of general sensitivity test showing the effectiveness of LLLT in a short period (0 to 48 h) (**a**), after 2 weeks (**b**), 1 month (**c**), and 2 months after BSSRO (**d**)

Four studies have examined the effectiveness of LLLT on recovery from neurosensory disturbance using general sensitivity tests dominantly via the visual analog scale (VAS) [17, 18, 33, 34]. Based on the result of the analysis, it can be observed that the LLLT is not effective in a short period (0 to 48 h) after surgery (WMD 0.11, 95% CI: −0.19 ~ 0.41, *I*^2^ = 0%, no heterogeneity). Also, the results of meta-analysis show that the application of LLLT is significantly effective in 2 weeks (SMD 1.07, 95% CI: 0.47 ~ 1.68, *I*^2^ = 0%, no heterogeneity), 1 month (SMD 0.97, 95% CI: 0.54 ~ 1.40, *I*^2^ = 0%, no heterogeneity), and 2 months (WMD 0.82, 95% CI: 0.54 ~ 1.09, *I*^2^ = 0%, no heterogeneity) after BSSRO. Also, the promising effects of the LLLT are seen in the period of 6 to 24 months after surgery based on general sensitivity tests (*P* < 0.05) [19, 33] (Fig. 5).

### Other neurosensory tests

Thermal discrimination test is performed using a thermometer, hot pipe, or hot gutta-percha. Studies showed no significant difference in terms of thermal sensitivity test after LLLT in treatment group/side compared to placebo even over 2 months after surgery (*P* > 0.05) [19, 32, 33]. Also, studies demonstrated that LLLT can reduce pain at 1 month after surgery based on the VAS test (*P* < 0.05) [19, 34]. LLLT was significantly effective in augmenting directional discrimination (a test in which patients should detect the path of a nylon filament) at 1 month after LLLT (*P* < 0.05) [33, 34]. Contact (touch) detection (a test in which a patient should assess the direction of fine brush stroked across the affected area) and pinprick test (was defined as the patient’s ability to find out the sharp needle touching the affected skin) have shown no significant difference in terms of neurosensory recovery between the treatment and control groups/sides (*P* > 0.05) [33]. However, Esmaeelinejad et al. showed a significant difference at 12 months after treatment intervention in terms of contact detection and pinprick tests between treatment and placebo groups [32].

## Adverse effects

No remarkable complications related to LLLT have been described in all included RCTs.

## Discussion

This meta-analysis was aimed to evaluate the effectiveness of LLLT on augmenting recovery of neurosensory disturbance (NSD) of the inferior alveolar nerve caused by BSSRO surgery. The results of the analysis revealed that generally, low-level laser therapy (LLLT) improves the recovery from neurosensory disturbance caused by BSSRO. This conclusion is supported by the literature [13, 17, 18, 20, 31–35]. BSSRO leads to some complications such as inferior alveolar nerve (IAN) damage, classified as neuropraxia, axonotmesis, and neurotmesis [20]. Neurotmesis is a rare condition and needs microsurgical repair [34]. Commonly, neurosensory deficit after BSSRO is a combination of axonotmesis and neuropraxia [20, 36]. Third molar extraction is recommended 6 months before surgery due to the increased risk of the bad split that may exacerbate nerve damage [13].

Frequently affected areas of nerve damage are the lower lip and chin which have been raised in most studies [17]. The literature has identified low-level laser therapy as a bio-modulatory tool which can be a promising technique for recovery from NSD after BSSRO [37]. The recovery of the nerve damage with help of LLLT can be measured by subjective or objective examinations. Examples of subjective tests include 2-point discrimination, general VAS score for sensitivity, and thermal discrimination and objective tests mainly include the following items: trigeminal evoked potential, electrical thermography, electromyography, and mental nerve blink reflex [11]. Agbaje et al. concluded that the most common approach to assess neurosensory deficits in literature was to use subjective assessments [9]. The studies included in our meta-analysis were no exception and most of them had used subjective tests.

Based on the meta-analysis, it seems that promising results cannot be expected from the LLLT in a short period (0 to 48 h) after surgery. All the included studies fully confirm the results of the meta-analysis that in a short interval after treatment intervention, LLLT does not improve recovery from NSD [13, 18, 33, 34]. Even within 2 weeks after surgery, it seems no statistically significant difference is considerable according to the two-point discrimination test, although the general sensitivity test shows a significant difference. The mechanisms proposed for nerve repair may not be effective at these intervals due to the lack of time for bio-modulation [17].

Here, according to the meta-analyses, maybe the promising results can mainly be observed at the period more than 1 month after surgery. On the other hand, LLLT has no significant effect on some aspects of neurosensory recovery, such as thermal sensation [33, 34]. It seems that thermal receptors return to their previous condition more speedily than those of other receptors [34].

In terms of limitations in this study, we can refer to the lack of reliable objective tests, shortness of follow-up after treatment, and a great amount of heterogeneity in protocols used for LLLT among studies. Also, the low number of appropriate studies for meta-analysis was another hindrance that we faced. We recommend that studies with longer post-treatment follow-up periods and larger sample sizes to be carried out to enrich the available literature. In addition, the lack of a standard protocol for LLLT after this type of surgery is felt. Moreover, it is critical to consider the individual characteristics of each patient’s anatomy.

## Conclusions

The results of the meta-analysis suggest that totally 8 to 10 sessions of low-level laser therapy is a safe method that accelerates the recovery of IAN neurosensory disturbances in orthognathic surgeries. Further high-quality clinical trials with longer follow-up periods and larger sample sizes are needed to increase the strength of evidence and to confirm the efficacy of LLLT for recovery from neurosensory disorders after orthognathic surgery.

## Data Availability

The datasets generated and/or analyzed during the current study are not publicly available due but are available from the corresponding author on reasonable request.

## Abbreviations

BSSRO: Bilateral sagittal split ramus osteotomy
LLLT: Low-level laser therapy
IAN: Inferior alveolar nerve
NSD: Neurosensory disturbance
VAS: Visual analog scale
SMD: Standard mean difference
WMD: Weighted mean difference
